# Clinical Observations on Operations for Congenital Clefts in Gums, Lips, and Palate in Early Infancy

**Published:** 1890-09

**Authors:** Thomas H. Manley

**Affiliations:** New York


### A Few Clinical Observations on Operation for Congenital Clefts in the Gums, Lips and Palate, in Early Infancy.

BY THOMAS H. MANLEY, A.M., M.D., OF NEW YORK.

Read in the Section of Dental and Oral Surgery, at the Forty-first Annual Meeting of the American Medical Association at Nashville, Tenn., May, 1890.

As defects of development or deficiencies of growth in and about the buccal and nasal cavities, of varied phases and degrees, are extremely common, it might seem that anything offered, of a practical character, in connection with them, would be of special interest and value to dental surgeons, whose domain, in my judgment, should include the whole bony framework of the alveolar arches, the palatine vault, and the cavernous sinuses of the nares, covered and nourished by the membrane of Schneider.

In the process of evolution, from birth, up through the varied stages of bodily growth, it is interesting to note, with the eruption of the teeth, the period of suckling, and the acquisition of speech, the many delicate and complicated changes in adjustment, structure and function, observed along the outlines and walls of the oral portal; that opening through which must pass all the aliments, the respiratory gases and phonetic sounds, and on the harmonious contour of which so much depends, for expression, character and beauty. My experience has led me to the conclusion that, with the solitary exception of a fissure in the soft palate, the time at which many congenital defects in this region may be most successfully and rapidly remedied, is when the infant is very young, and during the early stages of development.

During the past eighteen months, I have done five operations for harelip, in all of which the gum was fissured, and the floor of the nose opened through. When I have had the opportunity of selection, I have always preferred the *earliest possible* period after the delivery of the mother.

I am convinced that, in the vast majority of these cases, the deformity is due, not to any deficiency of structure proper, but to a defective arrangement; and acting on this theory, my line of action has been to replace the divided parts before ossification is well advanced, while those thin cartilaginous shells and tufts are readily flexible, and yield under moderate pressure.

I have observed in every instance, that with a limited osteoclasis, and reposition of the separated osseous segments, the division, in the soft parts, almost wholly disappears. The tension being removed, the refreshening and union of the cleft labia were always a matter of ease and simplicity. My patients' ages ranged from a few days to 6 months.

The first of this series had a fissure extending through the lips, gum and hard palate, and was 9 days old when I operated. After a preliminary osteoclasis, I wired the separated edges of the alveolar borders of the gum with silver wire; then carrying the scalpel along the dividing line of the mucous and cutaneous convexity of the lip, separated and later closed them, after the method of Maurice Collis, of Dublin, *i. e.*, not the *smallest particle* of tissue was sacrificed.

The wound healed kindly, and in ten days all the superficial sutures were removed. The baby now took the nipple, and continued thereafter to nurse without the least difficulty. In three weeks' time I removed the deep, osseous, metallic sutures.

My next cases, two in number, I operated on in the summer—early in August. One baby was 4 days old, the other 8 days.

With the first, the cleft was through all the parts, hard and soft, into the pharynx, on one side only, the left; the alveolar ridge, nasal septum and palate being forced outward, and away from the left for a considerable distance. Owing to the great chasm, and for want of some sort of central support, the columnar nasi had fallen in; which gave the nose a flattened out, shapeless form.

This case, though accompanied with the greatest degree of distortion, was the most simple of management. Acting precisely on the same principles as the preceding, the divided segments of the arch were brought together, and the vermillion borders of the lips closed. This case did well, except that two of the superficial sutures ulcerated through, requiring another trivial operation on the thirtieth day. Four day after the operation I was obliged to be absent from the city for two weeks, and it was during this time, for want of proper attention, that the accident happened.

The third had a double ridge, spreading widely open both cavities. The nasal and buccal cavities were thrown into one. The intermaxillary tuft was crowded forward, and curved in an upward direction. The little one, owing to the want of any sort of suction apparatus, swallowed with great difficulty. Every time liquids were swallowed, the head had to be well thrown back, and fairly poured into the stomach-tube. In proceeding with the operation in this case, for the first time, I had to use the chisel and mallet, in order to bring the bones into position, doing an osteotomy on both sides. I entered the chisel just posterior to the canine fossa, on a line with the pterygoid process of the temporal bone. This enabled me to replace the bony segments and complete the operation in the usual manner. The advanced state of ossification in this case was most singular. But we sometimes meet with an analogous state of the cranial bones, in parturition, which makes labor difficult or impossible.

As this infant was operated on at the same time as the second, from the same causes primary union was not wholly complete, and some later repairing had to be done. After this, the little one did well, the wound completely closing, when he was taken down with cholera infantum and died. Case four was identical, anatomically with the one third narrated, except that owing to the child's advanced age, several changes had taken place. He was 6 months old. The central segment was pushed much further forward; the maxillaries on both sides had advanced in similar directions to such a degree that their anterior margins barely touched each other; in this manner lifting up and throwing forward the piece attached to the perpendicular plate of the ethmoid, and giving it a most hideous and snout-like appearance.

The parents kept the mouth almost constantly covered, not only to conceal the deformity, but to prevent the almost constant drooling from the open vent. It was quite apparent at the first glance, without examining in detail, that there was a formidable case before us for operation. The time for dentition had arrived, the powers of locomotion, observation and reason were becoming manifest; the parts had firmly ossified, and adapted themselves to their deformed position; hence an operation which contemplated the division, resection, or crushing of those parts, must be attended with much loss of blood, and great shock. This, indeed, seemed so clear to me, that I fully advised the parents of the great dangers in the way, and made up my mind to divide the measures for relief into two or three operative stages. Prof. Jarvis has recommended me to the mother of the child, and as they had come a great distance they were determined to take the chances, rather than have the boy grow into manhood with this terrible deformity.

My plan of attack was to first content myself with an osteoclasis; to forcibly crowd back and fracture the maxillaries, then, if the septum refused to yield, remove a wedge of it at the point of junction of the vomer with the ethmoidal plate. This would permit me to place the intermaxillary segment where it belonged and as the tuft always contains four teeth, the central and lateral incisors, it was indispensable to save it at all hazards, for without it, the shape of the mouth is forever lost, and as the opposing jaws would so widely deviate, that the use of the teeth as triturators or grinders of the food would be forever lost. If I succeeded well with the preliminary operation and the baby safely survived it, I intended later to do a superficial plastic and uranoplastic operation. Very much to my surprise and gratification, on moderate pressure, the lateral walls of the upper jaw gave away sufficiently to allow me to bring closer, and fit into the breach made the projecting intermaxillary tuft. Before, however, this came into position, I had to remove a small U-shaped piece of the bony septum. Now I passed a heavy riveted piece of wire through the margin of the upper maxillary, through the centre of the intermaxillary, transfixing and permanently adjusting its free end, near the border of the opposite segment. This step solidly and firmly fixed the whole alveolar arch; besides gave form and expression to that part, where before there was none. As this was so readily and rapidly executed, I was tempted to complete the plastic on the soft parts at the same time, which I did. The infant was under chloroform something more than two hours.

He rallied well, and his prospect seemed excellent till the evening of the next day, when his temperature went up to 103° at midnight, when I was called and found him in a violent convulsion. He came out of it a moment after I entered. Now 3 grs. of quinine were given him *per rectum.* The next morning his fever had disappeared, and his general condition was good. His wound did well. On the sixth day I removed two of the superficial sutures on each side. Primary union had taken place except at the point where the base of the nostrils were closed. Here there had been infection from the nasal mucus. I introduced a fresh suture, and provided against future infection by stuffing both apertures with iodoform gauze.

When the integument was well healed, and the three separated segments had fused together, I removed the deep girder of wire from the bony structures underneath. The mother and child left for home just two weeks from the day of operation.

Fifth case. Male child 8 weeks old. This case was also one in which there was a double fissure dividing everything from the lip backward through the gums, hard and soft palate, except along the under convex margin of the nasal openings, where a very narrow ridge of integument connected the nasal cartilage with apex of the cleft lip.

The intermaxillary segment was displaced forward to a considerable extent, but not to such a degree as in the other cases, owing to this very diminutive but useful remnant of cutaneous tissue, moderating and restraining a further outward advance of it.

Though the baby could not draw the mammary gland, he was able to nurse the bottle through the rubber nipple, but, owing to the inability of perfecting the intermittent vacuum in suction, a considerable quantity leaked out from above through the nares.

The child was fairly healthy, though not very vigorous. This baby was operated on Wednesday, May 13, and through the courtesy of the family physician, Dr. Geo. S. Lamoree, of Highland, New York, and by consent of the parents, it was done at my usual weekly clinic at the Harlem Hospital. I commenced operative procedures, *in this instance*, by cutting off the immediate and largest blood supply to the parts, through which the blood must pass, by ligating both coronaries at each labial commissure with the temporary transfixion ligature. Then the field of operation was thoroughly washed and drenched with the mercuric chloride solution and every source of possible contamination shut off by light, soft sponges.

Finding that the bony septum was somewhat resistant to backward pressure, I cut a thin, wedge-shaped piece away from it, when I was able to place the central tuft where nature intended it should permanently lodge. The remainder of the operation was completed in the same manner as all the preceding.

The clinical, though only one side to be considered, in connection with these cases, is full of interest and is, I think, second only in importance to operative procedures.

In every one of these cases there was either a history of heredity or maternal impression. In case 1, maternal fright was clear and unmistakable. In case 2, the father's labio-nasal column was clearly defectively developed at birth, as evidenced by the immobile, contracted movement of the upper lip. In cases 3 and 5, maternal impressions were unquestionable, as both mothers' immediately preceding children were similarly marked. In case 4, a cousin of the mother had this deformity. In both of these, operations had been done, but neither of the babies survived. In my series there were three females and two males. In all nursing was impossible until the breach in the tissues had been closed in. Certainly, in every instance, a most repulsive deformity would remain if the chasm were left open and all labial and lingual articulate sounds would be impossible of utterance.

The nasal secretions, especially of a catarrhal character, discharging into the mouth and blending with the ailments, must inevitably befoul and taint the stomach, constantly provoking indigestion or diarrhœa.

The fundamental principles to be observed in the operation for relief of this deformity, as I perform it, while simple, must yet be observed to the letter, or our efforts will fail to attain what we aim for.

1. To operate as soon as possible after birth, provided the infant is healthy.

2. To do a preliminary osteoclasis when the bony framework in involved.

3. Hæmorrhage must be absolutely suppressed when possible.

4. Antiseptics must be applied with the utmost diligence, as on the latter we almost solely depend for primary union of the soft parts.

5. In trimming or preparing the margins for adaptation or adjustment, it is of cardinal importance that not the slightest morsel of tissue be sacrificed; for though, by free paring or slicing away, the mechanical part may seem more simple, and the immediate cosmetic effect will be better, yet, it must be borne in mind that condensation and contraction follow every sort of mutilation of the integuments, no matter how slight, and further because, in the process of evolution of the teeth, the parts will be put on the stretch to such an extent as to totally obliterate the apparent good effects of the operation, rendering speech difficult and painful and the ready opening of the mouth impossible.

6. Unless under exceptional circumstances, the surgeon should decline to operate unless his patient remain within convenient distance, so that he can be seen once or twice a day or oftener, during the first week—in fact, until solid union of the superficial parts is solid and complete.

As the region of operation in these cases is in immediate contact with escaping mucus and saliva, milk, soups or other liquid aliments, it is extremely difficult to preserve the line of suture wholly free from pathogenic germs. To provide against this as far as possible, it is necessary to close in everything hermetically and to stuff both nares well from the bottom with iodoform gauze, hence I use liquid collodion, applying several coats of it before adjusting other dressings.

Unfortunately, in some cases the tender cuticle is intolerant to antiseptics and a painful dermatitis develops, when the dressings must be promptly changed, or the deeper connective tissues become involved, with a plastic exudate and swelling which will seriously disturb or prevent healing. In one of my cases every sort of suture excited ulceration along its tract, and I had to depend on adhesive plaster to complete the sealing of the part. The constitutional treatment is of vital importance.

The infant is always in pain for the first day or two and needs the *unceasing* attention of the mother. No *nurse* can take her place. I invariably give a small dose of calomel immediately after operation and sufficient of the camphorated tr. of opium to keep the infant quiet, for violent crying puts the parts on a dangerous strain; besides, I believe convulsions are less apt to follow when the reflexes are not blunted by the analgesic action of the narcotic.

I allow the usual food to the infant when not nursing, though in two instances the patient was able to take the nipple of the mother after the tenth day. In every case, with a nursing child, the milk should be drawn away and fed to the baby until union is complete, when it can be taken in the usual way.

---

Note.—In case 5, three days before I left for the Nashville meeting, I operated. When I returned I found everything broken down; sepsis had developed, rendering everything a total failure. I now divided the oral muscles on the right side and went over the procedure again; but succeeded only in fixing the intermaxillary bone and getting union of the soft parts on one side. As the baby was very frail and inclined to tuberculosis, I had the parents take him home. I will, later, when his general health is improved, endeavor to unite the cleft on this side. In the meantime I have, in presenting this brochure, been desirous of having my professional brethren see just precisely and honestly what has been done; the bad or unfavorable results as well as the successes.

---

A Disappointed Dentist.—A dentist received compensation from an aristocratic cliant which he deemed insufficient, and remarked ironically: "Sir, you probably intend this money for my servant's fee." Whereat the patient retorted: "No, it is for both of you."

